# Malignant Change in an Epidermal Cyst Over Gluteal Region

**DOI:** 10.4103/0974-2077.79195

**Published:** 2011

**Authors:** Ashok Y Kshirsagar, Sanjitsingh R Sulhyan, Shradha Deshpande, SV Jagtap

**Affiliations:** *Department of Surgery, Krishna Institute of Medical Sciences University, Karad, Maharashtra, India*

**Keywords:** Epidermal cyst, squamous cell carcinoma, gluteal region

## Abstract

A 72-year-old male presented with a large ulceroproliferative lesion over left gluteal region. After histopathological confirmation of squamous cell carcinoma, the lesion was excised with wide margins. Further histopathological study of the excised specimen revealed the growth arising from an epidermal cyst. Malignant change is a rare, but wellknown complication occurring in an epidermal cyst. The mainstay of treatment consists of wide excision of cancerous lesion with primary reconstruction of the defect.

## INTRODUCTION

Malignant transformation of epidermal cysts is a rare occurrence. We hereby report a case of development of squamous cell carcinoma in a longstanding epidermal cyst over left buttock. The patient underwent wide excision of lesion with primary skin grafting of the defect. The case is reported because of its rarity.

## CASE REPORT

**Figure 1:**
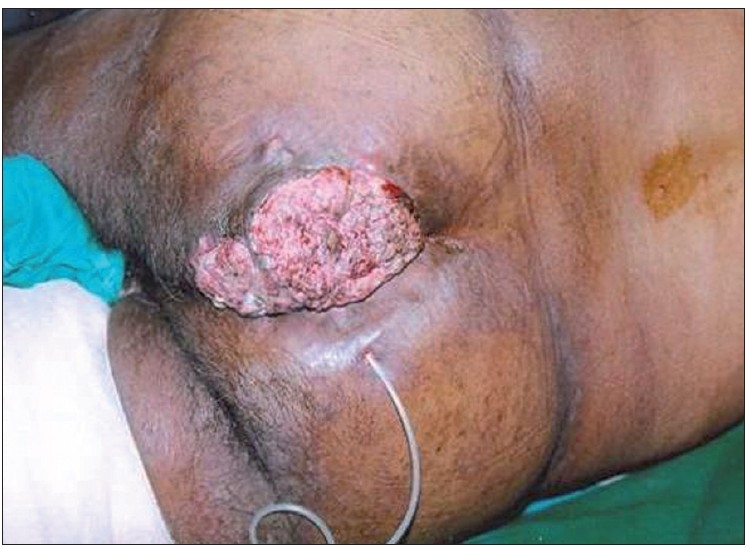
Photograph showing ulceroproliferative lesion over left gluteal region with chronic discharging sinus over right gluteal region

**Figure 2:**
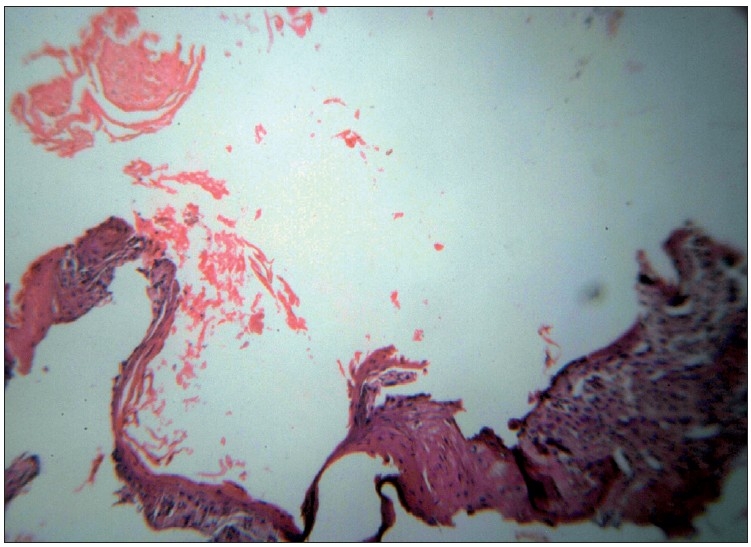
Photomicrograph showing epidermal cyst with lumen filled with keratinous material. (H and E, ×100)

**Figure 3:**
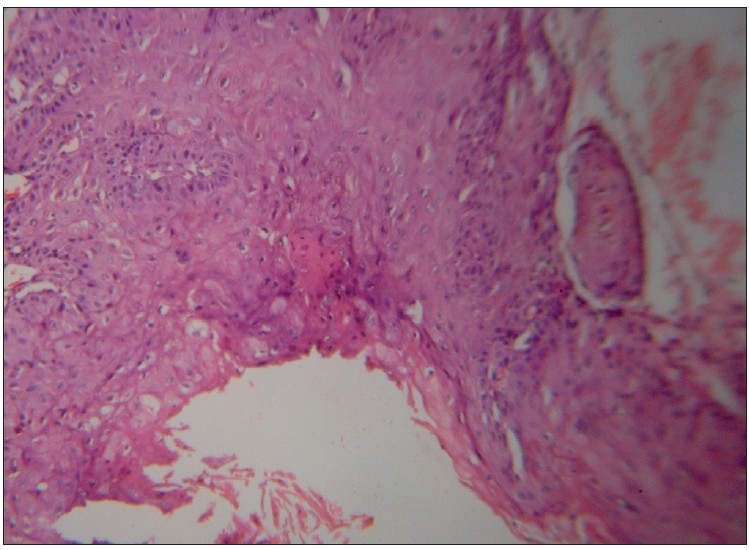
Photomicrograph showing cyst wall with areas of squamous cell carcinoma with foci of invasion. (H and E, ×400)

A 72-year-old man came with complaints of a large ulceroproliferative lesion over left gluteal region since 15 days. The patient had a history of small nodular swelling at the same site since 10 years. The initial small swelling gradually increased in size and ulcerated 15 days back after which the lesion grew rapidly. The patient also gave history of a chronic discharging sinus over opposite buttock. The discharge from the sinus primarily contained purulent material and occasionally blood. There was no history of rectal bleeding. There was no clinically significant lymphadenopathy. The patient’s systemic examination revealed no abnormality and his routine investigations were within normal limits. Perrectal examination and sigmoidoscopy did not reveal any abnormality. On local examination, an oval ulceroproliferative lesion of about 10×7 cm was noted on left gluteal region near the natal cleft.[Figure 1] The growth was not fixed to underlying muscle and had everted edges. Area surrounding the growth was indurated. The floor of the lesion was covered with blood-stained purulent discharge. The lesion was non-tender. A chronic discharging sinus was found on right buttock. Purulent discharge could easily be expressed from the sinus. Additional three to four nodular swellings of 12 cm of diameter were found over both the buttocks. Histopathologically, multiple sections from tissue showed ulcerative lesion and deeper tissue with thick-walled epidermal cyst [Figure 2]. The cyst was lined by squamous epithelium at areas forming squamous hyperplasia, dysplasia; and invasive squamous cell carcinoma which was composed of neoplastic cells arranged in small sheets, clusters and masses [Figure 3]. The cells were showing atypical features such as variation in size, shape, nuclear hyperchromasia, pleomorphism, absence of intracellular bridges, individual cell keratinisation and increased mitotic figures. Inflammatory cells were seen between the tumour cells. The biopsied sinus tract turned out negative for malignancy but exhibited changes consistent with epidermal cyst. Thus, histopathological diagnosis was given as squamous cell carcinoma with low malignant potential in an epidermal cyst.

The patient subsequently underwent wide excision of the lesion. Split thickness skin grafting was done over the defect. Simultaneously transverse loop colostomy was done to prevent soiling of grafted operative wound. The chronic discharging sinus tract was also excised and histopathologically no malignancy was detected in the sinus tract.

## DISCUSSION

Epidermal cysts are slowly growing, elevated, round firm intradermal or subcutaneous tumours found most commonly on face, scalp, neck and trunk. These cysts arise spontaneously in hair-bearing areas and are thought to be related to follicular infundibulum.[1] A few case reports indicate rare occurrence of these cysts in non-follicular regions like palms or soles. Traumatic implantation of epidermis into the dermis or subcutis is the cause of formation of epidermal cysts in such unusual sites.[2]

Histopathologically, epidermal cysts have a wall composed of true epidermis, as seen on the skin surface and in the infundibulum of hair follicles. In young epidermal cysts, several layers of squamous and granular cells can usually be recognized. In older epidermal cysts, the wall is markedly atrophic, either in some areas or in the entire cyst. In such cysts, only one or two layers of greatly flattened cells may form the wall. The cyst is filled with horny material arranged in laminated layers.

Rupture of an epidermal cyst releasing its contents into the dermis entails a considerable foreign- body reaction with numerous multinucleated giant cells. This leads to disintegration of cyst wall and formation of a keratingranuloma. Alternatively, it can also result in pseudocarcinomatous proliferation in remnants of the cyst wall, which can simulate a squamous cell carcinoma.[3] In pseudocarcinomatous proliferation, the squamous cells are usually well differentiated and the changes like atypia, individual cell keratinisation, nuclear hyperchromasia are minimal or absent[4]; in pseudocarcinomatous proliferation, there is permeation of inflammatory cells. In our case, there was cellular atypia, invasion of tumour in stroma and inflammatory cell infiltration was seen in between the tumour cell clusters which confirmed the diagnosis of squamous cell carcinoma. Development of true squamous cell carcinoma in epidermal cysts is a rare event and very few such cases have been reported in the literature so far.[5,6] Generally, squamous cell carcinoma occurring in epidermal cysts is of low malignant potential.[7] This was found in our patient also. The differential diagnosis was proliferating trichelemmal (pilar) tumour which forms wellcircumscribed multilobulated mass in the dermis composed of keratinocytes with dense eosinophilic cytoplasm. On low-power microscopic findings, tumour has sharply demarcated borders and lacks stromal invasion and within tumour, central cystic change may be seen.

Wide excision of the cancerous lesion with primary reconstruction of the defect forms the mainstay of treatment. Reconstruction using free latissimus dorsi flap, a buttock rotation flap and a posterior thigh rotation flap have been reported.[5] But in our patient as the tumour did not invade underlying musculature, primary reconstruction using a split thickness skin graft was done. In rare cases of advanced malignancy, where radical treatment is not possible, other treatment modalities are proposed. These modalities comprise topical treatment with 5 Fluorouracil [5 FU] ointment and systemic chemotherapy using intraarterial injection of bleomycin and 5 FU.[8] True efficacy of such modality remains to be evaluated.
